# Prolonged Diuretic and Renoprotective Effects of a Xanthone Obtained from *Garcinia achachairu* Rusby in Normotensive and Hypertensive Rats

**DOI:** 10.1155/2021/5510053

**Published:** 2021-04-20

**Authors:** Luísa Nathália Bolda Mariano, Thaise Boeing, Valdir Cechinel Filho, Rivaldo Niero, Arquimedes Gasparotto Junior, Luisa Mota da Silva, Priscila de Souza

**Affiliations:** ^1^Programa de Pós-graduação em Ciências Farmacêuticas (PPGCF), Núcleo de Investigações Químico-Farmacêuticas (NIQFAR), Universidade do Vale do Itajaí (UNIVALI), Rua Uruguai, 458, Centro, 88302-901, Itajaí, Brazil; ^2^Laboratório de Farmacologia Cardiovascular (LaFaC), Faculdade de Ciências da Saúde, Universidade Federal da Grande Dourados, R. João Rosa Góes, 1761, CEP 79825-070, Dourados, Brazil

## Abstract

The previous study showed that 1,5,8-trihydroxy-4′,5′-dimethyl-2H-pyrano(2,3 : 3,2)-4-(3-methylbut-2-enyl) xanthone (TDP) obtained from *Garcinia achachairu* Rusby (Clusiaceae) branches induces acute diuresis in normotensive (NTR) and spontaneously hypertensive rats (SHR) after 8 h of the experiment. In complementarity, the present study evaluated the prolonged diuretic and renoprotective effects of TDP in both NTR and SHR. The animals received, once a day, oral treatment with TDP (0.1 mg/kg), hydrochlorothiazide (10 mg/kg), or vehicle (VEH; 10 mL/kg). At the end of 7 days, the urine, blood, and kidney samples were collected for biochemical and histological analyzes. The urinary volume of both NTR and SHR after 7 days of treatment with the TDP was significantly increased, associated with augmented urinary electrolyte excretion levels. The treatments did not modify the urinary pH values nor the parameters analyzed in plasma (Na^+^, K^+^, Cl^−^, and Ca^2+^). Concerning the renal analyzes, when compared with the VEH-treated NTR group, while the activity of the enzymes catalase (CAT) and N-acetyl-*β*-D-glucosaminidase (NAG), as well as nitrite levels, were increased, the generation of lipid hydroperoxides and the activity of the enzyme myeloperoxidase (MPO) were unaltered. On the other hand, the activities of superoxide dismutase (SOD) and glutathione *S*-transferase (GST) and the levels of reduced glutathione (GSH) in kidney homogenates of the SHR group were decreased. However, TDP augmented the levels of GSH and GST activities and reduced the levels of nitrite and the activities of CAT and MPO, when compared with VEH-treated only SHR. Besides, the treatment with TDP alleviated the morphological changes of the renal corpuscle region of SHR. Together, these results revealed the prolonged diuretic effect of TDP and their renal protective effect by improving the antioxidative capacity.

## 1. Introduction

Diuretic drugs are used to treat cardiovascular and kidney disorders [1, 2]. Diuretics' main action is to increase the concentration of Na^+^ and water in the renal tubules [1, 3], resulting in diuresis [4]. There are five diuretic classes, and each type differs in efficacy, mechanism of action, and location of effects in the nephron [5], so it is common to associate different categories [6]. Diuretic classes commonly used as antihypertensives are loop diuretics, thiazide-type diuretics, and potassium-sparing agents [7].

Despite the variety of diuretics, their use is associated with the risk of developing several adverse effects, such as electrolyte disturbances, ventricular arrhythmias [8], increased risk of acquiring on-set diabetes [9], sexual dysfunction, gynecomastia [3], and ototoxicity [2]. As a result, medicinal plants and isolated compounds have been widely studied as possible alternatives to complement current therapy or reveal potential new study molecules. Some medicinal plants that are already used in folk medicine for the treatment of cardiovascular and renal disorders have scientific studies proving their diuretic effects, such as *Tropaeolum majus* L. [10], *Achillea millefolium* L. [11], *Maytenus ilicifolia* Mart ex Reissek [12], *Scutia buxifolia* Reissek [13], *Echinodorus grandiflorus* (Cham & Schltdl) [14], *Bauhinia forficata* Link [15], and *Leandra dasytricha* (A. Gray) Cong. [16].

Recently, we described the diuretic effect of the methanolic extract and fractions obtained of branches from *G. achachairu* Rusby (Clusiaceae) [17, 18], which is a plant native of Bolivia, popularly known as “achachairu” [19]. Phytochemical study permitted to isolate the 1, 5,8-trihydroxy-4′,5′-dimethyl-2H-pyrano (2,3 : 3,2)-4-(3-methylbut-2-enyl) xanthone (TDP), which showed a significant diuretic effect in rats after a single-dose treatment [17, 18]. Xanthones are polyphenolic compounds, and their structures are recognized for their importance in discovering new active compounds [20]. Indeed, several biological activities have been described for xanthones, including anti-inflammatory, antioxidant [21], cardioprotective [22], diuretic [17, 23], and renal protective [24] effects.

Given the above and considering that TDP has already shown an acute diuretic action in our previous publication, this study aimed to investigate the diuretic and renoprotective effects of TDP after a dose-repeated treatment in both normotensive and hypertensive rats.

## 2. Materials and Methods

### 2.1. Xanthone Isolation

**Figure 1:**
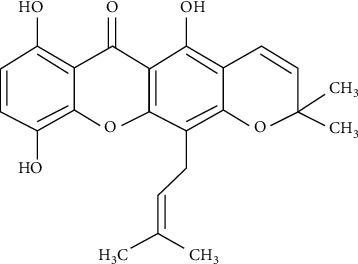
Molecular structure of the 1,5,8-trihydroxy-4′,5′-dimethyl-2H-pyrano(2,3 : 3,2)-4-(3-methylbut-2-enyl) xanthone (TDP).

Details of the isolation of 1,5,8-trihydroxy-4′,5′-dimethyl-2H-pyrano(2,3 : 3,2)-4-(3-methylbut-2-enyl) xanthone (TDP) (Figure 1) obtained from branches of *G. achachairu* are described by Mariano et al. [25].

### 2.2. Animals

Female Wistar normotensive (NTR) and spontaneously hypertensive rats (SHR) of 3-4 months old were used in this study. The animals were provided by Universidade do Vale do Itajaí (UNIVALI) and were maintained in a controlled laboratory environment (12 h light/dark cycle and 22 ± 2°C), with free access to food and water. All methodologies were approved by the Ethical Committee for the Care and Use of animals of UNIVALI (authorization 028/17).

### 2.3. Prolonged Diuretic Activity Assay

The rats were randomly distributed into groups of 6–8 animals, and each group was treated daily, with vehicle (VEH; water plus 1% tween; 10 mL/kg, p.o), hydrochlorothiazide (HCTZ; 10 mg/kg, p.o), or TDP compound (0.1 mg/kg, p.o). The animals were individually allocated in metabolic cages, the urine was collected, and the volume was recorded every day for 7 days. The cumulative urine volume was calculated to bodyweight and expressed as mL/100 g. At the end of the experiment, the organs (heart, kidney, liver, lung, and spleen) were removed and weighed. Blood samples were collected for biochemical analysis. Besides, renal tissue samples were collected for the assessment of the tissue redox state and histological analysis.

### 2.4. Biochemical and Tissue Redox State Evaluation

Blood samples were examined for the content of electrolytes, creatinine, urea, aspartate aminotransferase (AST), alanine aminotransferase (ALT), and nitrite [17, 26]. The urine samples were analyzed for electrolytes excretion (Na^+^, K^+^, Ca^2+^, and Cl^−^), pH, and osmolality. Osmolality was calculated using the formula described by Bhasin and Velez, 2016 [27].

The oxidative stress analysis followed the methodologies detailed and described by De Almeida et al. [28]. Briefly, the renal tissue was homogenized in potassium phosphate buffer (200 mM with pH 6.5; 1 : 3 weight/volume). This homogenate was used to measure the levels of reduced glutathione (GSH) and lipid hydroperoxides (LOOH). Posteriorly, the homogenate was centrifuged (20 min at 9000 g). The supernatant was used to determine the activity of the enzymes superoxide dismutase (SOD), catalase (CAT), and glutathione *S*-transferase (GST) [28]. The pellet was used to determine the activity of the enzymes myeloperoxidase (MPO) [28] and N-acetyl-*β*-D-glucosaminidase (NAG) [29].

### 2.5. Histological Analysis

Kidney tissue was fixed in ALFAC solution (85% ethanol, 10% formaldehyde, and 5% acetic acid), and after 24 h, this tissue was dehydrated (alcohol and xylene), embedded in paraffin, and stained with hematoxylin/eosin (H&E). The material was examined using a stereo microscope with a magnification of 40x.

### 2.6. Statistical Analysis

The program GraphPad Prism version 7.00 for Mac (GraphPad Software, La Jolla, CA, USA) was used for the statistical analysis. The results were expressed as mean ± standard error of the mean (S.E.M.) of 6–8 animals per group. The differences between means were determined by one-or two-way analysis of variance (ANOVA) followed by Dunnett's multiple comparisons test. The value was considered statistically significant when the value of *p* is less than 0.05.

## 3. Results and Discussion

Bolda Mariano et al. [18] described the acute diuretic effect of the xanthone TDP (Figure 1), in which a single oral dose of 0.1 mg/kg was able to induce diuresis and increase of the urinary electrolytes excretion in both normotensive (NTR) and hypertensive (SHR) rats. Herein, to better understand the renal effects of TDP, especially in a dose-repeated treatment, the present study was performed.

The first set of results showed that the TDP (0.1 mg/kg) increased the urine volume of both NTR and SHR compared to VEH (Table 1). The treatment with HCTZ (10 mg/kg), a thiazide-type diuretic [30], as expected, was effective in the VEH group. The treatment with HCTZ increased the renal excretion of Na^+^, K^+^, and Cl^−^. The treatment with TDP increased Na^+^ excretion in NTR and SHR, confirming the compound's ability to induce diuresis [1, 3]. However, while TDP increased K^+^ excretion in NTR, the same was not detected when urine samples from SHR were analyzed. Indeed, it is necessary to be careful when using drugs that cause an increase in urinary K^+^ excretion due to the risk of developing hypokalemia, which is very common with the use of K^+^-depleting diuretics [31]. Besides, TDP was not able to increase Cl^−^ excretion neither in NTR nor SHR. HCTZ treatment, as expected, decreased the excretion of Ca^2+^ [31], while the compound TDP was also able to decrease Ca^2+^ levels in the urine. Diuretic drugs that can reduce urinary Ca^2+^ excretion are interesting to be used by patients with osteoporosis [32] or as a prophylactic treatment for kidney stones [33]. Osmolarity values were increased in the treated groups compared to the VEH animals, which was expected, since this parameter reflects the amount of ion excretion in the urine. Finally, the urinary values of pH were unaltered.

Additionally, the results obtained with plasma analysis are shown in Table 2. No statistically significant changes were found in the content of electrolytes (Na^+^, K^+^, Cl^−^ and Ca^2+^), uric acid, creatinine, urea, AST and ALT in the different experimental groups. However, nitrite levels, an indirect marker of NO production [34], were lower in SHR animals treated with VEH than VEH-treated NTR. The treatment with TDP was not able to increase the levels of nitrite in the plasma. This dataset shows that this xanthone probably does not have its diuretic and natriuretic effects related to the nitric oxide (NO) production. However, it is worth mentioning that in our previous study, indomethacin (a cyclooxygenase inhibitor) precluded TDP-induced diuresis [18], so we can suggest that the mechanisms responsible for the effects of xanthone presented here could involve direct vasodilator actions on the renal vascular bed.

The animals' weight, water, and food intake showed no differences during the 7 days of the experiment (data not shown). Besides, the weight of the kidneys and heart differed between the NTR groups and SHR treated with VEH (i.e., reduced kidney weight and increased cardiac weight in the SHR group—data not shown), which was expected, since the characteristics of SHR lineage are associated with ventricular hypertrophy, cardiac hyperplasia, and kidney damage [35]. Moreover, these results have already been described in previous studies [26, 28]. The treatment with HCTZ or TDP did not cause any changes in these tissues, suggesting that 7 days of treatment were not enough to reverse the damage already established by hypertension. Besides, the TDP did not cause any alteration in the weight of the other organs (i.e., liver, lung, and spleen—data not shown).

**Figure 2:**
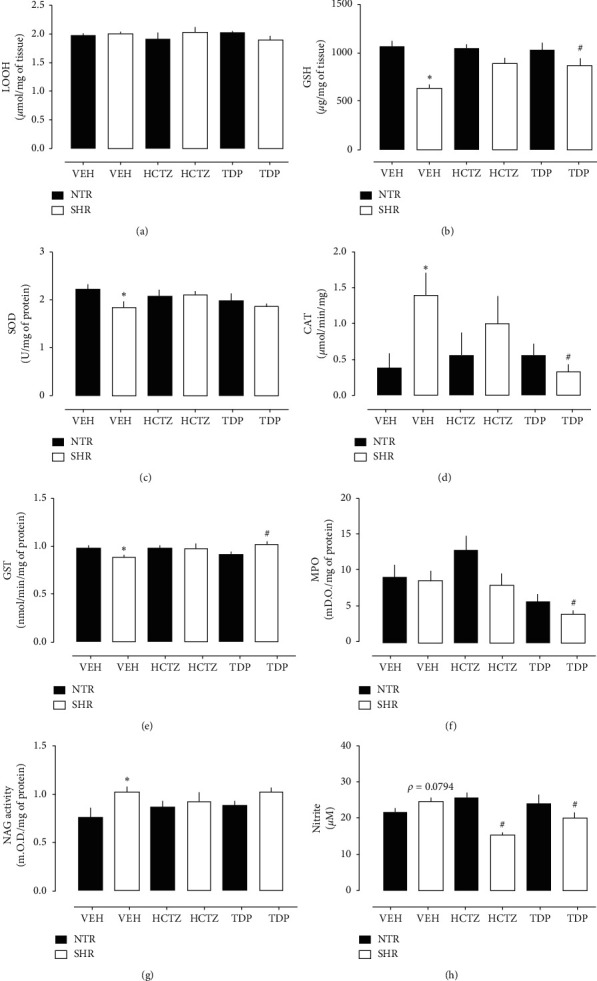
Effect of TDP on renal markers of oxidative stress, endogenous antioxidants factors, and cell biomarkers after 7 days of treatment in rats. (a) Lipid hydroperoxides (LOOH) content, (b) reduced glutathione (GSH) levels, (c) superoxide dismutase (SOD) activity, (d) catalase (CAT) activity, (e) glutathione S-transferase (GST) activity, (f) myeloperoxidase (MPO) activity, (g) N-acetyl-*β*-D-glucosaminidase (NAG) activity, and (h) nitrite levels in kidney samples collected from normotensive rats (NTR) and spontaneously hypertensive rats (SHR). The results show the mean ± S.E.M. of 6–8 animals per group. Statistical analysis was performed using one-way analysis of variance (ANOVA) followed by Dunnett's multiple comparisons test. ^∗^*p* < 0.05 when compared with the respective VEH group. #*p* < 0.05 when compared with the VEH-treated SHR group. TDP, 1,5,8-trihydroxy-4′,5′-dimethyl-2H-pyrano(2, 3 : 3, 2)-4-(3-methylbut-2-enyl) xanthone.

Oxidative stress and the deficiency of NO may be present in hypertension or renal disease [36], and the class of xanthones is known to have antioxidant effects [21]. For this reason, we investigated the involvement of the antioxidant system in the kidneys and the possible renal protector effect of TDP. First, the levels of lipid hydroperoxides (LOOH) were measured since lipid peroxidation is related to some disorders and can trigger a variety of oxidants, which can lead to cell dysfunction and tissue damage [37], in addition to indirectly indicating the oxidative stress in the tissue [38]; however, there was no difference between the groups (Figure 2(a)). It is believed that when triggering tissue damage, the defense system itself begins to act to repair the injury [39]; however, we cannot rule out the hypothesis of other cell damage that did not cover in this study. Regarding the reduced glutathione (GSH) levels in renal tissue (Figure 2(b)), which is an antioxidant biomolecule abundant in the body [40], in SHR treated with VEH (635 ± 35.46 *μ*g/mg of tissue), GSH levels were reduced by 40.54% compared to NTR treated with VEH (1068 ± 54.06 *μ*g/mg of tissue), and the treatment with TDP was able to reverse this value, reaching almost baseline levels. In sequence, we analyzed the enzymes SOD, CAT, and GST, as shown in Figures 2(c)–2(e), respectively. The animals of the SHR group treated with VEH showed decreased SOD and GST activities and increased CAT activity when compared to the NTR group treated with VEH. Briefly, these results demonstrated that the antioxidant system defense is lower in SHR animals treated with VEH only. On the other hand, TDP partially restored the antioxidant system (i.e., decreasing CAT activity and increasing GST activity), suggesting a possible antioxidant effect of this compound on renal tissue.

Additionally, we analyzed the effect of TDP on MPO and NAG activities in renal tissue, respectively (Figures 2(f) and 2(g)). The MPO is a biomarker of cell infiltration, mainly neutrophils, and a marker of acute inflammation in the tissue [41]. Likewise, NAG is an indicator of cell infiltration highly specific for macrophages [42]. Unlike neutrophils, macrophages remain in the tissue longer and are among the cells involved in chronic inflammation [43]. In this group of experiments, the results obtained show that the MPO activity did not change between the SHR and NTR groups treated with VEH only (Figure 2(f)). On the other hand, the NAG activity was higher in the SHR group treated with VEH than the NTR group treated with VEH only (Figure 2(g)), suggesting that the disease already well installed in the body; cells of the chronic inflammatory process are generally present. However, TDP treatment did not change NAG activity in renal tissue than the VEH group, although it reduced MPO activity in the SHR group. These data suggest that TDP may have an anti-inflammatory effect related to reducing neutrophil infiltration; however, further experiments are needed to confirm this effect. According to literature data, the class of xanthones can present an anti-inflammatory potential [21, 44].

NO is involved in several physiological processes in the kidneys, including diuresis and natriuresis [36]. Our kidney sample results reveal no differences in the nitrite content between the SHR and NTR treated with VEH only, a result similar to that found by Almeida et al. [28]. Interestingly, the SHR groups treated with HCTZ and TDP showed a decreased value of nitrite in the renal tissue (Figure 2(h)), a result that can be directly linked to the local inflammatory response [45].

**Figure 3:**
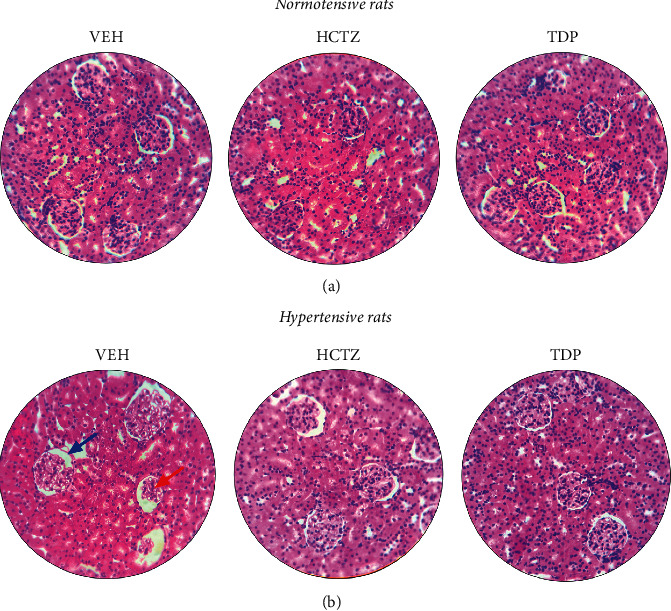
Representative images of renal tissue morphology stained by hematoxylin and eosin (H&E). (a) Normotensive rats and (b) spontaneously hypertensive rats. The blue and red arrows indicate Bowman's capsule region and the renal glomerulus, respectively. VEH,  vehicle (water plus 1% tween). HCTZ, hydrochlorothiazide, and TDP, 1,5,8-trihydroxy-4′,5′-dimethyl-2H-pyrano(2, 3 : 3, 2)-4-(3-methylbut-2-enyl) xanthone.

Finally, the histological results of the kidneys obtained from SHR animals treated with VEH (Figure 3(b)) showed a disruption of the mesangial space, an increase in the glomerular size, and an increase in the thickening of the Bowman capsule when compared with the histology of VEH-treated NTR (Figure 3(a)). The changes found in the SHR group treated with HCTZ and TDP are less evident when compared to the group treated with VEH. Besides, histological analysis of renal tissue did not show any changes in NTR animals treated with HCTZ or TDP in NTR (Figure 3(a)). This result is significant because it shows that after 7 days of treatment, there was no glomerular or tubular damage induced by TDP, supporting the absence of adverse effects.

## 4. Conclusion

The prolonged treatment with TDP-induced significant diuretic and natriuretic effects, restored the imbalance of the antioxidant system in the renal tissue, and mitigated hypertensive rats' renal damage, revealing an important renoprotective effect.

## Figures and Tables

**Table 1:** Urinary parameters were measured after 7 days of treatment with TDP in normotensive and hypertensive rats.

Groups	Urine volume (mL/100 g)	pH	Osmolarity (mOsm/kg H_2_O)	Na^+^ (mmol/L)	K^+^ (mmol/L)	Cl^−^ (mmol/L)	Ca^2+^ (mg/dL)
NTR							
VEH (10 mL/kg)	9.75 ± 0.77	8.39 ± 0.25	362.0 ± 13.06	129.9 ± 8.15	44.57 ± 3.04	335.3 ± 3.45	14.26 ± 0.53
HCTZ (10 mg/kg)	23.13 ± 0.44^∗^	8.69 ± 0.08	877.4 ± 53.51^∗^	306.3 ± 13.86^∗^	98.09 ± 4.59^∗^	360.0 ± 2.77^∗^	6.26 ± 0.90^∗^
TDP (0.1 mg/kg)	14.91 ± 0.69^∗^	8.75 ± 0.06	520.2 ± 24.66^∗^	172.7 ± 5.51^∗^	65.49 ± 3.29^∗^	324.2 ± 7.92	10.33 ± 0.55^∗^

SHR							
VEH (10 mL/kg)	9.44 ± 0.73	8.58 ± 0.15	399.5 ± 25.05	122.1 ± 8.31	40.45 ± 3.43	316.1 ± 6.61	8.39 ± 0.95
HCTZ (10 mg/kg)	17.52 ± 1.24^∗^	8.73 ± 0.02	747.8 ± 31.76^∗^	272.4 ± 12.91^∗^	76.08 ± 8.43^∗^	358.0 ± 7.66^∗^	3.67 ± 0.35^∗^
TDP (0.1 mg/kg)	15.66 ± 0.91^∗^	8.61 ± 0.17	507.1 ± 41.52^∗^	171.3 ± 12.93^∗^	46.74 ± 3.73	317.0 ± 7.13	5.33 ± 0.80^∗^

The results show the mean ± S.E.M. of 6–8 animals per group. Statistical analysis was performed using one-way analysis of variance (ANOVA) followed by Dunnett's multiple comparisons test. ^∗^*p* < 0.05 when compared with the respective VEH group. NTR, normotensive rats; SHR, spontaneously hypertensive rats; VEH, vehicle (water plus 1% tween), HCTZ, hydrochlorothiazide, and TDP, 1,5,8-trihydroxy-4′,5′-dimethyl-2H-pyrano(2,3 : 3, 2)-4-(3-methylbut-2-enyl) xanthone.

**Table 2:** Plasma parameters after treatment with TDP for 7 days in normotensive and spontaneously hypertensive rats.

Groups	Na^+^ (mmol/L)	K^+^ (mmol/L)	Cl^−^ (mmol/L)	Ca^2+^ (mg/dL)	Uric acid (mg/dL)	Creatinine (mg/dL)	Urea (mg/dL)	AST (U/L)	ALT (U/L)	Nitrite (*μ*M)
NTR										
VEH (10 mL/kg)	116.6 ± 9.40	3.09 ± 0.43	224.3 ± 2.79	4.79 ± 0.04	4.61 ± 0.09	0.39 ± 0.01	36.30 ± 2.66	47.63 ± 1.27	46.91 ± 6.16	67.26 ± 3.39
HCTZ (10 mg/kg)	127.5 ± 8.50	4.12 ± 0.46	229.4 ± 4.85	4.36 ± 0.11	4.67 ± 0.17	0.45 ± 0.02	30.45 ± 2.69	45.00 ± 5.10	40.11 ± 3.06	56.48 ± 3.09
TDP (0.1 mg/kg)	117.9 ± 5.88	3.56 ± 0.23	220.8 ± 1.60	4.86 ± 0.16	4.73 ± 0.08	0.38 ± 0.01	35.49 ± 1.39	41.01 ± 1.77	36.60 ± 1.95	60.93 ± 3.62

SHR										
VEH (10 mL/kg)	123.3 ± 3.93	3.21 ± 0.87	225.2 ± 2.66	4.97 ± 0.19	4.52 ± 0.18	0.38 ± 0.01	31.91 ± 1.20	47.38 ± 1.85	46.06 ± 2.79	54.71 ± 3.97^#^
HCTZ (10 mg/kg)	113.9 ± 7.88	2.85 ± 0.28	222.7 ± 0.57	4.47 ± 0.25	4.60 ± 0.20	0.36 ± 0.01	37.40 ± 0.78	36.27 ± 1.48	47.81 ± 3.47	62.65 ± 2.99
TDP (0.1 mg/kg)	106.3 ± 2.78	2.15 ± 0.41	227.7 ± 3.58	5.10 ± 0.18	4.60 ± 0.09	0.38 ± 0.01	35.46 ± 1.82	48.45 ± 3.99	40.55 ± 2.60	52.40 ± 3.59

The results show the mean ± S.E.M. of 6–8 animals per group. Statistical analysis was performed using one-way analysis of variance (ANOVA) followed by Dunnett's multiple comparisons test. *∗p* < 0.05 when compared with the respective VEH group. #*p* < 0.05 when compared with the VEH-treated SHR group. NTR, normotensive rats; SHR. spontaneously hypertensive rats; VEH, vehicle (water plus 1% tween); HCTZ, hydrochlorothiazide; TDP, 1,5,8-trihydroxy-4′,5′-dimethyl-2H-pyrano(2,3 : 3,2)-4-(3-methylbut-2-enyl) xanthone.

## Data Availability

The data used to support the findings of this study are available from the corresponding author upon request.

## Abbreviations

ALT: Alanine aminotransferase
AST: Aspartate aminotransferase
CAT: Catalase
GSH: Reduced glutathione
HCTZ: Hydrochlorothiazide
H & E: Hematoxylin and eosin
iNOS: Inducible nitric oxide synthase
LOOH: Lipid hydroperoxides
MPO: Myeloperoxidase
NAG: N-acetyl-*β*-D-glucosaminidase
NO: Nitric oxide
NTR: Normotensive rats
SHR: Spontaneously hypertensive rats
SOD: Superoxide dismutase
GST: Glutathione S-transferase
TDP: 1,5,8-Trihydroxy-4′,5′-dimethyl-2H-pyrano(2,3 : 3,2)-4-(3-methylbut-2-enyl) xanthone
VEH: Vehicle.
